# Transcription factor organic cation transporter 1 (OCT-1) affects the expression of porcine Klotho (*KL*) gene

**DOI:** 10.7717/peerj.2186

**Published:** 2016-07-14

**Authors:** Yan Li, Lei Wang, Jiawei Zhou, Fenge Li

**Affiliations:** Key Laboratory of Pig Genetics and Breeding of Ministry of Agriculture & Key Laboratory of Agricultural Animal Genetics, Breeding and Reproduction of Ministry of Education, Huazhong Agricultural University, Wuhan, PR China

**Keywords:** KL gene, OCT-1, Pig, MARC0022311

## Abstract

Klotho (KL), originally discovered as an aging suppressor, is a membrane protein that shares sequence similarity with the *β*-glucosidase enzymes. Recent reports showed Klotho might play a role in adipocyte maturation and systemic glucose metabolism. However, little is known about the transcription factors involved in regulating the expression of porcine *KL* gene. Deletion fragment analysis identified KL-D2 (−418 bp to −3 bp) as the porcine *KL* core promoter. MARC0022311SNP (A or G) in KL intron 1 was detected in Landrace × DIV pigs using the Porcine SNP60 BeadChip. The pGL-D2-A and pGL-D2-G were constructed with KL-D2 and the intron fragment of different alleles and relative luciferase activity of pGL3-D2-G was significantly higher than that of pGL3-D2-A in the PK cells and ST cells. This was possibly the result of a change in *KL* binding ability with transcription factor organic cation transporter 1 (OCT-1), which was confirmed using electrophoretic mobility shift assays (EMSA) and chromatin immune-precipitation (ChIP). Moreover, OCT-1 regulated endogenous *KL* expression by RNA interference experiments. Our study indicates SNP MARC0022311 affects porcine *KL* expression by regulating its promoter activity via OCT-1.

## Introduction

The Klotho (*KL*) gene encodes a membrane protein that shares a sequence similarity with the *β*-glucosidase genes and its product may function as part of a signaling pathway that regulates aging and morbidity in age-related diseases (Ko et al., 2013). Mutant mice lacking the *KL* gene shows multiple aging disorders and a shortened life span (Kuro-o et al., 1997). *KL*^−∕−^ mice have the pattern of ectopic calcification certainly contributed by the elevated phosphate and calcium levels (Hu et al., 2011; Ohnishi et al., 2009). KL also acts as a deregulated factor of mineral metabolism in autosomal dominant polycystic kidney disease (Mekahli & Bacchetta, 2013). Mice that lacked Klotho activity are lean owing to the reduced white adipose tissue accumulation, and are resistant to obesity induced by a high-fat diet (Ohnishi et al., 2011; Razzaque, 2012).

*KL* expression is regulated by thyroid hormone, oxidative stress, long-term hypertension and so on (Koh et al., 2001). Some transcription factors such as peroxisome proliferator-activated receptor gamma (PPAR-*γ*) also can regulate *KL* expression (Zhang et al., 2008). A double- positive feedback loop between PPAR-*γ* and Klotho regulates adipocyte maturation (Chihara et al., 2006; Zhang et al., 2008). Briefly, chromatin immuno-precipitation (ChIP) and gel shift assays find a PPAR-responsive element within the 5′-flanking region of human *KL* gene. Additionally, PPAR-*γ* agonists increases *KL* expression in HEK293 cells and several renal epithelial cell lines, while the induction is blocked by PPAR-*γ* antagonists or small interfering RNAs (Zhang et al., 2008). Furthermore, Klotho can induce PPAR-*γ* synthesis during adipocyte maturation (Chihara et al., 2006). However, little is known about the transcription factors involved in regulating the expression of porcine *KL* gene.

Several hundreds of thousands of porcine SNPs were discovered using next generation sequencing technologies, and Illumina Inc used these SNPs, as well as others from different public sources, to design a high-density SNP genotyping assay (Ramos et al., 2009). SNP MARC0022311 is one 64,232 SNPs on the Porcine SNP60K BeadChip. In our study, we detected MARC0022311 SNP in some pigs using 60K SNP chip and found that this SNP could affect the transcriptional regulation of *KLOTHO* gene. To investigate the transcriptional regulation of porcine *KL* gene, we identified the core promoter of porcine *KL* gene, analyzed its upstream regulatory elements and revealed that transcription factor OCT1 directly bound to the core promoter region of porcine *KL* gene and regulated its expression.

## Materials and Methods

### Ethics statements

All animal procedures were performed according to the protocols approved by the Biological Studies Animal Care and Use Committee of Hubei Province, PR China. Sample collection was approved by the ethics committee of Huazhong Agricultural University (No. 30700571 for this study).

### MARC0022311 polymorphism in pigs

Nineteen Landrace × DIV crossbred pigs were genotyped with the Porcine SNP60 BeadChip (Illumina) using the Infinium HD Assay Ultra protocol, which was conducted under the technical assistance by Compass Biotechnology Corporation. DIV was a synthetic dam line derived by crossing Landrace, Large White, Tongcheng or Meishan pigs. Raw data had high genotyping quality (call rate >0.99) and were analyzed with the GenomeStudio software.

### *In silico* sequence analysis

*KL* gene sequence ENSSSCG00000009347 was available on the ENSEMBL online website (http://asia.ensembl.org/index.html). We obtained the up-stream sequence of porcine *KL* gene for promoter prediction. The potential promoter was analyzed using the online neural network promoter prediction (NNPP) (http://www.fruitfly.org/seq_tools/promoter.html) and Promoter 2.0 prediction server (http://www.cbs.dtu.dk/services/Promoter/). Transcription factor binding sites were predicted using biological databases (BIOBASE) (http://www.gene-regulation.com/pub/programs.html) with a threshold of 0.90 and TFsearch with a threshold of 85 (Akiyama, 1995). Threshold represents minimum probability of predicted transcription factor.

### Cell culture, transient transfection and luciferase assay

The porcine kidney (PK) cells and swine testis (ST) cells obtained from China Center for Type Culture Collection (CCTCC) were cultured at 37 °C in a humidified atmosphere of 5% CO2 using DMEM supplemented with 10% FBS (Gibco).

Four *KL* promoter deletion fragments (KL-D1: −178 bp to −3 bp, KL-D2: −418 bp to −3 bp, KL-D3: −599 bp to −3 bp and KL-D4: −835 bp to −3 bp) were cloned into *pGL3-Basic* vector to determine the core promoter region. The plasmids contained pig *KL* intron 1 fragments (g.1474 A and g.1474 G) followed by KL-D2 promoter (−418 bp to −3 bp) were reconstructed, then transfected using lipofectamine 2000 (Invitrogen) into PK cells and ST cells. Plasmid DNA of each well used in the transfection containing 0.8 µg of *KL* promoter constructs and 0.04 µg of the internal control vector *pRL-TK* Renilla/luciferase plasmid. The enzymatic activity of luciferase was then measured with PerkinElmer 2030 Multilabel Reader (PerkinElmer).

### RNA interference

Double-stranded small interfering RNAs (siRNAs) targeting *OCT-1* were obtained from GenePharma. Cells were co-transfected with 2 µl of siRNA, 0.2 µg of reconstructed plasmids using Lipofetamine 2000™ reagent for 24 h. Transfection mixtures were removed, and fresh complete DMEM medium was added to each well. Finally, the enzymatic activity of luciferase was then measured with PerkinElmer 2030 Multilabel Reader (PerkinElmer).

### Quantitative real time PCR (qPCR)

qPCR was performed on the LightCycler^®^ 480 (Roche) using SYBR^®^ Green Real-time PCR Master Mix (Toyobo). Primers used in the qPCR were shown in Table 1. qPCR conditions consisted of 1 cycle at 94 °C for 3 min, followed by 40 cycles at 94 °C for 40 s, 61 °C for 40 s, and 72 °C for 20 s, with fluorescence acquisition at 74 °C. All PCRs were performed in triplicate and gene expression levels were quantified relatively to the expression of *β*-actin. Analysis of expression level was performed using the 2^−ΔΔ*Ct*^ method (Livak & Schmittgen, 2001). Student’s *t*-test was used for statistical comparisons.

**Table 1:** Primers and DNA oligos used in this study.

Primer	Primer sequence (5′-3′)	Amplicon length (bp)	Tm (°C)
5′-Bio of A (+)	GGTAATGTTGTAATAATGGCTAA		60
5′-Bio of A (−)	TTAGCCATTATTACAACATTACC		
cold probe of A (+)	GGTAATGTTGTAATAATGGCTAA		60
cold probe of A (−)	TTAGCCATTATTACAACATTACC		
cold probe of G (+)	GGTAATGTTGTAATAGTGGCTAA		60
cold probe of G (−)	TTAGCCACTATTACAACATTACC		
*KL_*ChIP_ PF	TGAAGACCACTGCTACACACTT		59
*KL_*ChIP_ PR	AGCAAACAGGTTTTGTGGAGC		
*KL*_ D1_ PF	*CGG***GGTACC**TTGTTGGATGTTTTGTTTGTCTAGCTAGC	193	58
*KL*_ D_ PR	*CG***ACGCGT**CCCTGTGAAGGCTTGTTT		
*KL*_ D2_ PF	*CGG***GGTACC**TATGAGGAGGTGGGTTGGCTAGCTAGC	433	59
*KL*_ D_ PR	*CG***ACGCGT**CCCTGTGAAGGCTTGTTT		
*KL*_ D3_ PF	*CGG***GGTACC**CACTTAACCTCTTATTCTTGAGTTACTAGCTAGC	614	59
*KL*_ D_ PR	*CG***ACGCGT**CCCTGTGAAGGCTTGTTT		
*KL*_ D4_ PF	*CGG***GGTACC**ACATAAAAGTTAGAAAATCAGAGAACTAGCTAGC	850	59
*KL*_ D_ PR	*CG***ACGCGT**CCCTGTGAAGGCTTGTTT		
*OCT1_ qPCR_ PF*	TGAACAATCCGTCAGAAACC	196	58
*OCT1_ qPCR_ PR*	TGAGCAGCAGCCTGTAAACT		
*KL_ qPCR_ PF*	ACCCGTATTTATTGATGGAGAC	173	57
*KL_ qPCR_ PR*	GGAACTTCATCTGAGGGTCTAA		
*KL_ intron1_ ChIP_ PF*	GCCGTAGATAATTGAAGC	130	50
*KL_ intron1_ ChIP_ PR*	TCTGTGGTAGCAAACAGG		
*KL_ intron2_ ChIP_ PF*	GCCAGTGTAAGGTGTTACC	114	51
*KL_ intron2_ ChIP_ PR*	ATTCTCCAAAGAAGACATACA		
*KL_ intron3_ ChIP_ PF*	CAAGATTGTACCGTGGAG	171	50
*KL_ intron3_ ChIP_ PR*	GGTCATTTGACATCATTCT		

**Notes.**

Protective bases and induced enzyme sites were in italic and bold, respectively.

### Western blotting

Western blotting was performed as described previously (Tao et al., 2014). Five µg proteins were boiled in 5 × SDS buffer for 5 min, separated by SDS-PAGE, and transferred to PVDF membranes (Millipore). Then, the membranes were blocked with skim milk and probed with anti-KL (ABclonal). *β*-actin (Santa Cruz) was used as a loading control. The results were visualized with horseradish peroxidase-conjugated secondary antibodies (Santa Cruz) and enhanced chemiluminescence.

### Electrophoretic mobility shift assays (EMSA)

Nuclear protein of PK and ST cells was extracted with Nucleoprotein Extraction Kit (Beyotime). The oligonucleotides (Sangon) corresponding to the OCT-1 binding sites of *KL* intron 1 (Table 1) were synthesized and annealed into double strands. The DNA binding activity of OCT-1 protein was detected by LightShift^®^ Chemiluminescent EMSA Kit (Pierce). Ten µg nuclear extract was added to 20 fmol biotin-labeled double stranded oligonucleotides, 0.1 mM EDTA, 2.5% Glycerol, 1× binding buffer, 5 mM MgCl_2_, 50 ng Poly (dI⋅dC) and 0.05% NP-40. In addition, control group added 2 pmol unlabeled competitor oligonucleotides, while the super-shift group added 10 µg OCT-1 antibodies (Santa Cruz). The mixtures were then incubated at 24 °C for 20 min. The reactions were analyzed by electrophoresis in 5.5% polyacrylamide gels at 180 V for 1 h, and then transferred to a nylon membrane. The dried nylon was scanned with GE ImageQuant LAS4000 mini (GE-Healthcare).

### Chromatin immunoprecipitation (ChIP) assay

ChIP assays were performed using a commercially available ChIP Assay Kit (Beyotime) as previously described (Tao et al., 2015). Briefly, after crosslinking the chromatin with 1% formaldehyde at 37 °C for 10 min and neutralizing with glycine for 5 min at room temperature, PK and ST cells were washed with cold PBS, scraped and collected on ice. Then, cells were harvested, lysed and treated by sonication. Nuclear lysates were processed 20 times for 10 s with 20 min intervals on ice water using a Scientz-IID (Scientz). An equal amount of chromatin was immune-precipitated at 4 °C overnight with at least 1.5 µg of OCT-1 antibody (Santa Cruz) and normal mouse IgG antibody (Millipore). Immune-precipitated products were collected after incubation with Protein A + G coated magnetic beads. The beads were washed, and the bound chromatin was eluted in ChIP elution buffer. Then the proteins were digested with Proteinase K for 4 h at 45 °C. The DNA was purified using the AxyPrep PCR Cleanup Kit (Axygen). The DNA fragment of OCT-1 binding sites in *KL* intron 1 was amplified with the specific primers (Table 1). The PCR procedure was executed with 36 rounds and in the linear range, ChIP assay had 3 biological replicates.

### Statistical analysis

Statistical analyses based on two-tailed Student’s t-tests were performed using the Statistical Package for the Social Sciences software. Significance was determined at a 95% confidence interval. All data were expressed as the mean ± standard deviation (S.D.).

## Results

### MARC0022311 status in pigs

MARC0022311 in *KL* intron 1 appeared a polymorphism (A or G) in 19 Landrace × DIV pigs, with 12 AA pigs and AG pigs genotyped using the Illumina PorcineSNP60 chip (Data S1) . The SNP (MARC0022311) in pig *KL* intron 1 was renamed as *KL* g.1474 A > G according to the standard mutation nomenclature (Den Dunnen & Antonarakis, 2000).

**Figure 1:**
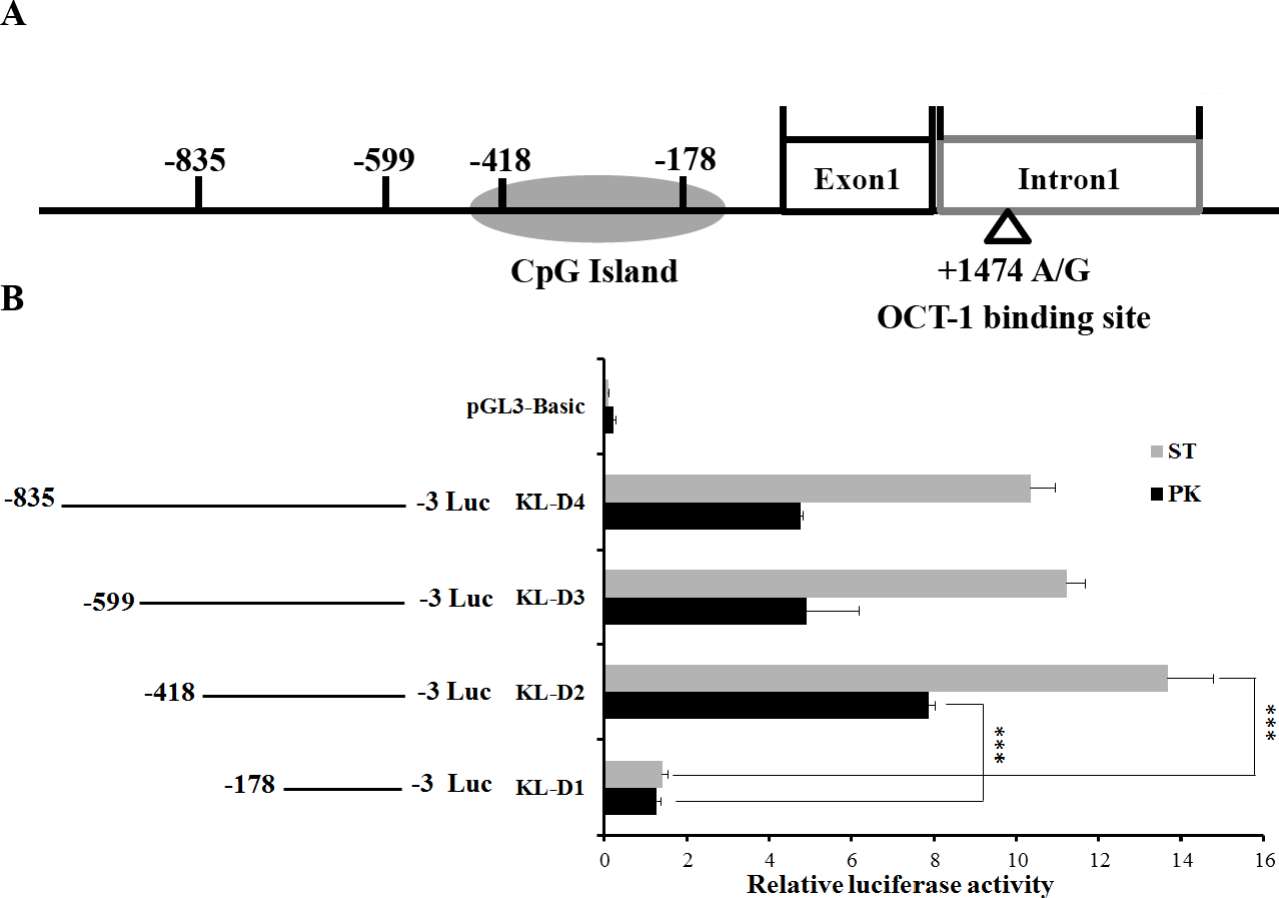
Deletion analysis of pig *KL* promoter. (A) Schematic diagram of *KL* promoter, MARC0022311 (*K*L g.1474 A > G) and OCT-1 binding site in intron 1. (B) Promoter activities of a series of deleted constructs determined by luciferase assay. The luciferase reporter construct containing the individual sequence KL-D1–KL-D4 (KL-D1:−178–−3 nt, KL-D2:−418–−3 nt, KL-D3:−599–−3 nt and KL-D4: −835–−3 nt) was transfected into ST cells and PK cells, and dual luciferase assays were performed 24 h after transfection. Firefly luciferase activity was normalized to the corresponding Renilla luciferase activity. Values are expressed as means ± SE of three replicates. ***P < 0.001.

### Identification of promoter region of the porcine *KL* gene

An 833 bp contig in 5′ flanking region of pig *KL* gene was obtained by PCR. To determine the promoter region, four promoter deletions (KL-D1, KL-D2, KL-D3 and KL-D4) were cloned into fluorescent vector based on the prediction of NNPP online software and Promoter 2.0 (Fig. 1A). Luciferase activity analysis in both PK and ST cells revealed that KL-D2 (−418 bp to −3 bp) was essential for its transcriptional activity and was defined as the *KL* promoter region (Fig. 1B).

### MARC0022311 SNP affects the *KL* expression

**Figure 2:**
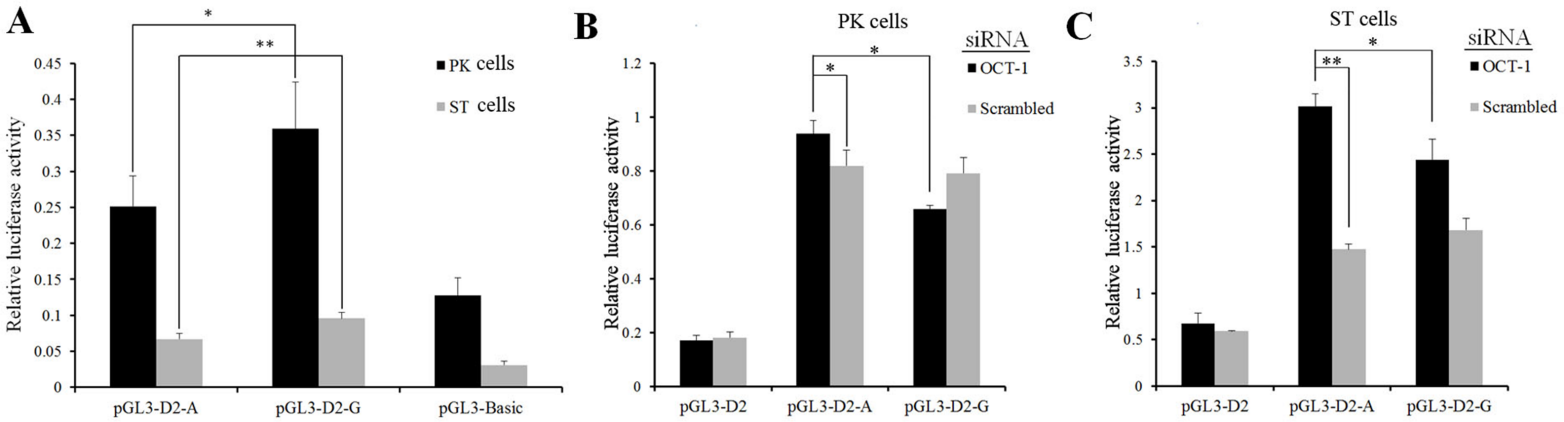
MARC0022311 in pig *KL* intron 1 affected promoter activity in PK and ST cells. (A) Luciferase assays of reporter constructs using pig KL-D2 promoter and intron 1 fragments (g.1474 A and g.1474 G). (B) Luciferase detection after co-transfection of *OCT-1* siRNA with *pGL3-D2-A* and *pGL3-D2-G* in PK cells. (C) Luciferase detection after co-transfection of *OCT-1* siRNA with *pGL3-D2-A* and *pGL3-D2-G* in ST cells. *P < 0.05. **P < 0.01. The pGL3-basic was used as the negative control.

Intron SNPs could not change the amino acid sequence, but might alter gene promoter activity by affecting the binding ability of transcription factors (Van Laere et al., 2003). The plasmids contained the wild-type A (g.1,474 A) or mutant G (g.1474 G) sequence followed by KL-D2 were named as pGL3-D2-A and pGL3-D2-G, respectively. Then recombinant DNA fragments were inserted in the downstream of the luc+ gene between the *Kpn*I and *Hin*dIII sites. Results showed that luciferase activity of *pGL3-D2-G* was significantly higher than *pGL3-D2-A* in both PK cells (*P* < 0.05) and ST cells (*P* < 0.01) (Fig. 2A), and indicated that MARC0022311 SNP changed the binding ability of certain regulatory elements affected KL promoter activity.

The SNP (MARC0022311) located in the first intron of *KL* gene (+1, 474 bp) was predicted to change the binding ability of OCT-1 by BIOBASE and TFsearch (Fig. S1). After silencing *OCT-1* using siRNAs in PK and ST cells, luciferase activity of *pGL3-D2-G* was significantly lower than *pGL3-D2-A* (*P* < 0.05) (Figs. 2B and 2C). Furthermore, compared with the negative control, the luciferase activity of *pGL3-D2-A* was significantly decreased (*P* < 0.05) (Figs. 2B and 2C). Thus, MARC0022311 regulated the promoter activity via OCT-1.

**Figure 3:**
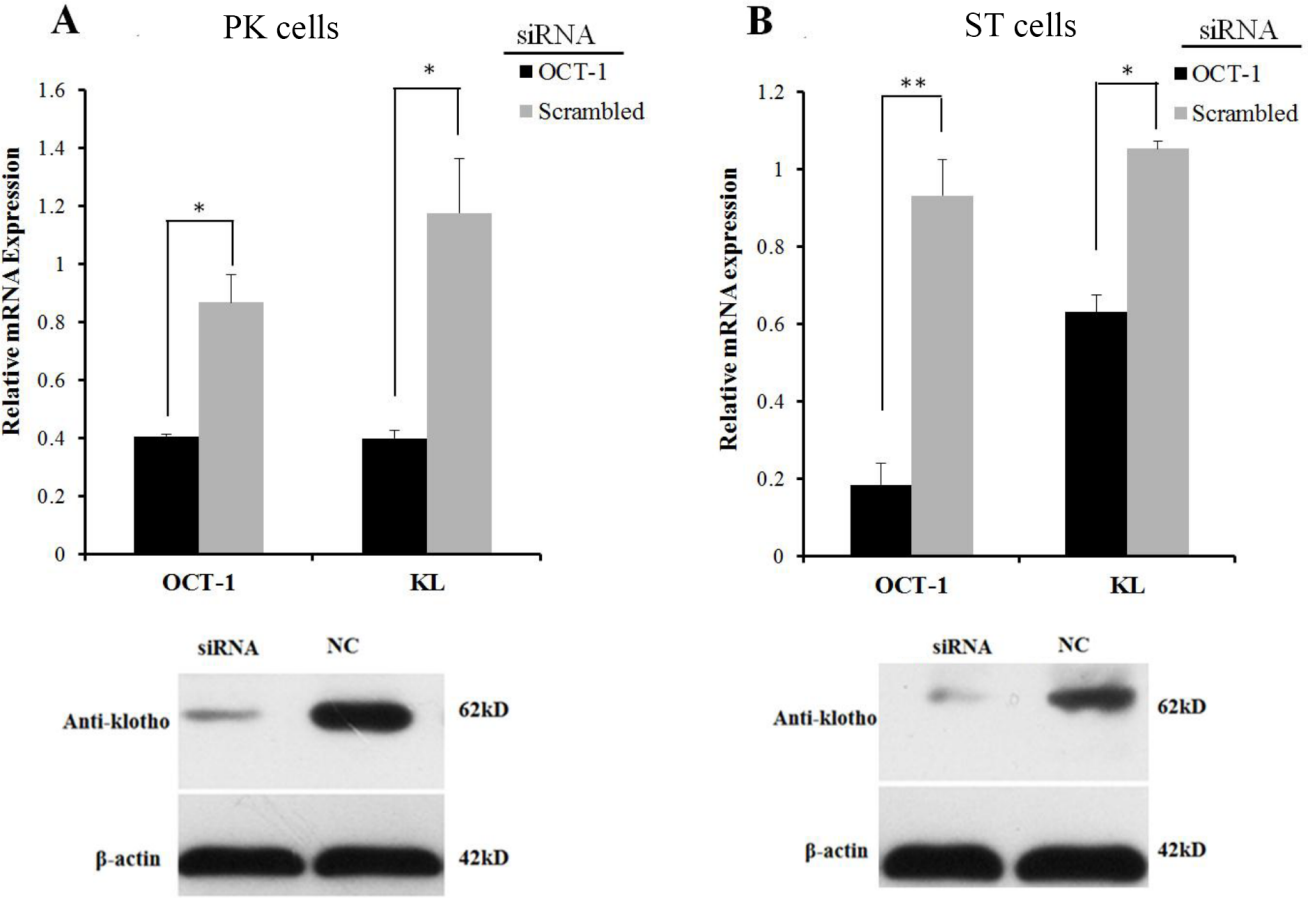
OCT-1 up-regulated *KL* expression by RNAi. (A) PK cells were treated with 2 µl *OCT-1* siRNA and 2 µl NC for 24 h. Knockdown of *OCT-1* was confirmed by qPCR. *KL* mRNA and protein expressions were analyzed by qPCR and Western blotting. (B) ST cells were treated with 2 µl *OCT-1* siRNA and 2 µl NC for 24 h. Knockdown of *OCT-1* was confirmed by qPCR analysis. *KL* mRNA and protein expressions were analyzed by qPCR and Western blotting. *P < 0.05. **P < 0.01. Relative mRNA expression was relative to the expression of *β*-actin.

However, inhibition of *OCT-1* expression significantly suppressed *KL* expression in PK and ST cells (*P* < 0.05) (Fig. 3), possibly because OCT-1 could stimulate *KL* expression by binding *KL* gene at other sites.

### Transcription factor OCT-1 binds to the *KL* intron 1 both *in vitro* and *in vivo*

**Figure 4:**
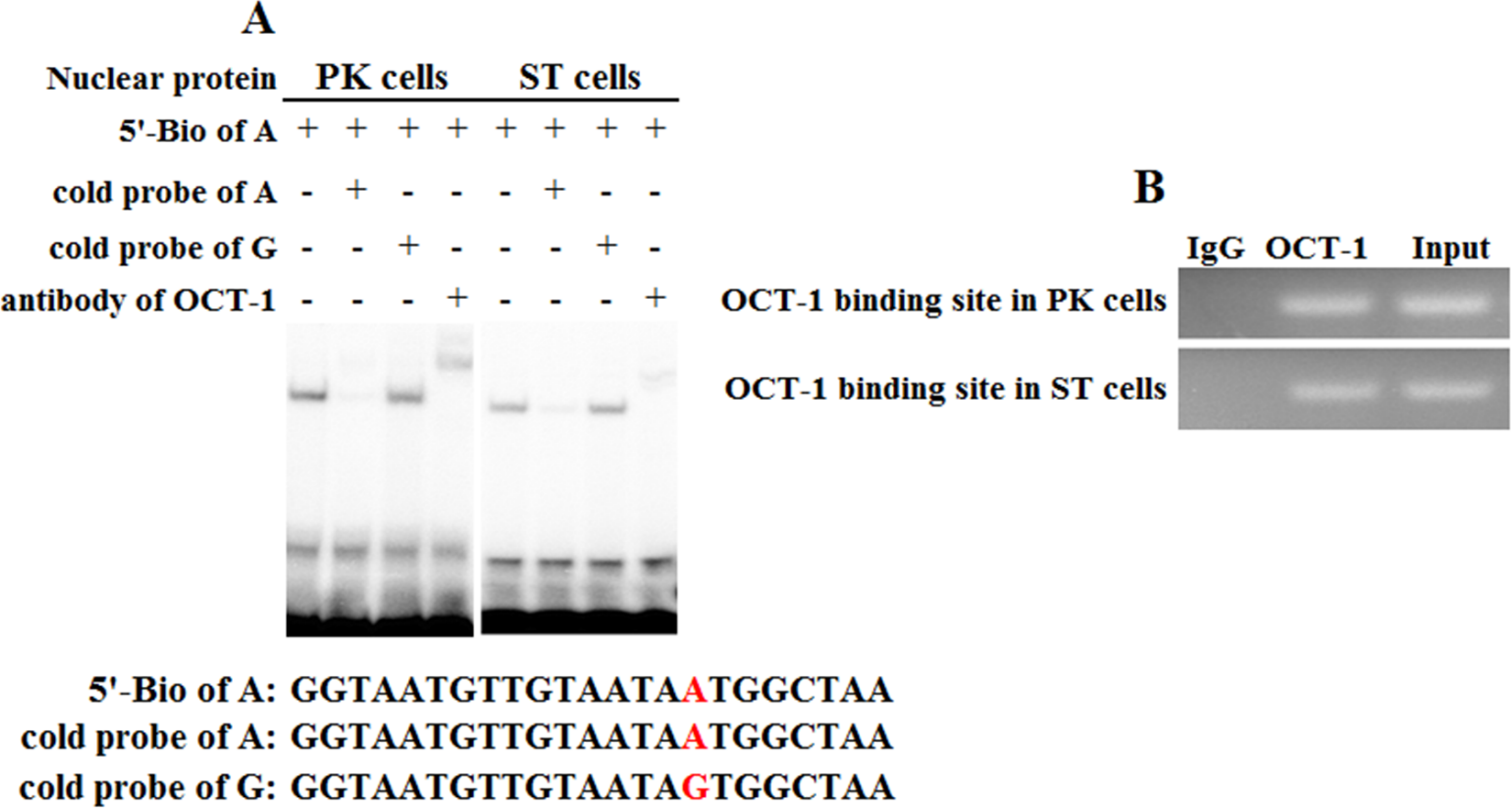
Binding of OCT-1 with *KL* intron 1 was analyzed by EMSA and ChIP. (A) The probe was incubated with nuclear extract in the absence or presence of 100-fold excess of various competitor probes (mutant or non-labeled probe) or anti-OCT-1. The specific super-shift (DNA-protein-antibody complex) bands were both observed in PK and ST cells. The sequences of various probes were demonstrated under the panel. (B) ChIP assay of OCT-1 binding to the *KL* intron 1 in PK cells and ST cells. The interaction of OCT-1 *in vivo* with *KL* intron region was determined by chromatin immunoprecipitation analysis. DNA isolated from immune-precipitated material was amplified by PCR to amplify *KL* fragement. Total chromatin was used as the input. Normal mouse IgG was used as a negative control.

To address whether KL intron 1 contained OCT-1 binding sites *in vitro*, we used two oligonucleotides (A allele and G allele oligonucleotides) as porcine OCT-1 probes in EMSA. EMSA revealed a highly specific interaction with allele A oligonucleotide, and a 100 fold excess of mutant allele G oligonucleotide could not outcompete the interaction (Fig. 4A). A super-shift was obtained when nuclear extracts from PK and ST cells were incubated with OCT-1 antibodies, providing further biochemical evidence for the presence of OCT-1 *in vitro* (Fig. 4A). We found the *KL* genotype at g.1474 A > G locus was AA in PK and ST cells by PCR-sequencing, indicating the endogenous binding of OCT-1 to *KL* in above two cell lines (Fig. S2). The chromatin was immune-precipitated using an OCT-1 antibody and DNA fragments of the expected size were used as a template to perform PCR amplification. ChIP analysis showed that OCT-1 interacted with *KL* intron 1 (Fig. 4B). These results showed that transcription factor OCT-1 bound to *KL* intron 1 both *in vitro* and *in vivo.*

## Discussion

*KL* gene encodes a type-I membrane protein that is related to beta-glucosidases (Ko et al., 2013). *KL* may function as part of a signaling pathway that regulates morbidity in age-related diseases such as atherosclerosis and cardiovascular disease, and mineral metabolism diseases such as ectopic calcification (Ko et al., 2013; Kuro-o et al., 1997; Hu et al., 2011; Ohnishi et al., 2009). Overexpression of *KL* in the preadipocyte 3T3-L1 cell line can induce expression of several adipogenic markers, including *PPARγ*, CCAAT/enhancer binding protein alpha (*C/EBPα*) and CCAAT/enhancer binding protein delta (*C/EBPδ*), and facilitate the differentiation of preadipocytes into mature adipocytes (Chihara et al., 2006). Eliminating *KL* function from mice results in the generation of lean mice with almost no detectable fat tissue, and induces a resistance to high-fat-diet-stimulated obesity (Razzaque, 2012; Ohnishi et al., 2011).

Here we found the SNP MARC0022311 located in KL intron 1 in the tested pigs (Data S1). A number of SNPs are proved to have major effects on the phenotypic variations (Markljung et al., 2009; Milan et al., 2000; Ren et al., 2011; Van Laere et al., 2003). Previous reports show that a G to A transition in intron 3 of porcine insulin-like growth factor 2 (*IGF2*) affects the binding of ZBED6 and significantly up-regulated *IGF2* expression in skeletal muscle (Markljung et al., 2009; Van Laere et al., 2003). We predicted the SNP MARC0022311 located in *KL* intron 1 could change the binding ability of transcription factors including OCT1 by BIOBASE and TFsearch online software (Fig. S1).

The Octamer-binding proteins (OCTs) are a group of highly conserved transcription factors that specifically bind to the octamer motif (ATGCAAAT) and closely related sequences that are found in promoters and enhancers (Zhao, 2013). OCT1 regulates the expression of a variety of genes, including immunoglobulin genes (Dreyfus, Doyen & Rougeon, 1987), *β*-casein gene (Zhao, Adachi & Oka, 2002), miR-451/AMPK signaling (Ansari et al., 2015), sex-determining region Y gene (Margarit et al., 1998), synbindin —related ERK signaling (Qian et al., 2015).

In the present study, the pGL3-basic was used as the negative control and inserted core promoter fragment with wild-type and mutant-type intron fragments (pGL3-D2-A, pGL3-D2-G). We wanted to check whether there was different in fluorescent activity between two kinds of plasmids (Fig. 2A). The pGL3-D2 was used as control and it did not contain intron fragments, and we wanted to verify whether the transcription factor binding to the inserted intron fragment was the activator or inhibitor (Figs. 2B–2C). The luciferase activity of pGL3-D2-G was significantly higher than pGL3-D2-A (Fig. 2A) and the following OCT-1 RNAi results showed that luciferase activity of pGL3-D2-G significantly decreased in the scrambled and the pGL3-D2-A in PK cells and ST cells (Figs. 2B–2C). The G allele missed one binding sites compared to the A allele (G allele had 2 binding sites, while A allele had 3 binding sites) (Fig. S1), and displayed a higher luciferase activity than A allele (Fig. 2A), suggesting that at this site OCT1 was a repressor. Therefore, we supposed that OCT-1 could bind to the first intron of *KL* when the SNP was allele A, and then depressed activity of *KL* promoter.

However, the expression of KL was significantly inhibited after silencing OCT-1. There were several OCT-1 binding sites in porcine KL intron 1 (36,324 bp in length) predicted by BIOBASE and TFsearch online software (Fig. S3A). ChIP analysis showed that OCT-1 interacted with all of three tested regions (1,395 bp to 1,525 bp, 14,322 bp to 14,436 bp, 30,970 bp to 31,141 bp) in PK cells (Fig. S3B). It was possible that there was a synergetic effect between the binding sites. Our aim was to detect the difference of OCT-1 binding sites within two alleles which might change KL gene expression. It was certain that A allele created a novel OCT-1 binding site within the flanking region of MARC0022311 SNP by online prediction (Fig. S1), which was further confirmed by dual-luciferase reporter assay system (Fig. 2) and EMSA (Fig. 4A). In consequence, we hypothesized that OCT1 could dimerise with the chromosome leading to stable binding of the DNA (Tommy et al., 2011; Zabet & Adryan, 2015). In our study, OCT1 could act as an activator and the presence of the third site in the A allele could disrupt the binding of the dimmer leading to lower activity of the A allele.

Klotho physiologically regulates mineral and energy metabolism by influencing the activities of fibroblast growth factors (FGFs) including FGF-2, FGF-19, FGF-23 and their receptors (FGFRs) (Guan et al., 2014; Razzaque, 2009; Wu et al., 2008). Taken together, KL exerts its function via OCT-1 - KL- FGF- FGFR pathway.

## Conclusions

In summary, SNP MARC0022311 affected OCT-1 binding ability with the *KL* promoter. And the *KL* promoter activity was significantly decreased in allele A of MARC0022311 compared with allele G. Our study indicated SNP MARC0022311 affected porcine *KL* expression by regulating its promoter activity via OCT-1.

##  Supplemental Information

10.7717/peerj.2186/supp-1 Figure S1 Transcription factor binding site prediction of the procine *KL* intron 1 containing MARC0022311 (KL g.1474 A>G) Quadrilateral frame indicated the substitutions and extra binding site of OCT-1. (A) Predicted by BIOBASE online software. (B) Predicted by TFserach online software.

10.7717/peerj.2186/supp-2 Figure S2 Genotyping results of MARC0022311 (A) PK cells. (B) ST cells. MARC0022311 was marked in gray backgound.

10.7717/peerj.2186/supp-3 Figure S3 OCT-1 binding sites in the porcine *KL* intron 1 (A) Frequency distribution of the predicted OCT-1 binding sites. X-axis indicated the length of the porcine *KL* intron 1 in bp. Y-axis was the frequency of the predicted OCT-1 binding sites. (B) ChIP analysis of three candidate OCT-1 binding sites (1,395 bp to 1,525 bp, 14,322 bp to 14,436 bp, 30,970 bp to 31,141 bp) in *KL* intron 1 in PK cells. Primers used for ChIP-PCR was shown in Table 1. Input and R were positive control, while IgG was the negative control.

10.7717/peerj.2186/supp-4 Data S1 Raw SNP genotyping results

## Additional Information and Declarations

### Data Availability

The following information was supplied regarding data availability: The raw data has been supplied as a Data S1.
