# Supplementary figures and images for: Transcription factor organic cation transporter 1 (OCT-1) affects the expression of porcine Klotho (KL) gene
Source: PeerJ. 2016 Jul 14;4:e2186. doi: 10.7717/peerj.2186 (PMC4950547; doi:10.7717/peerj.2186)

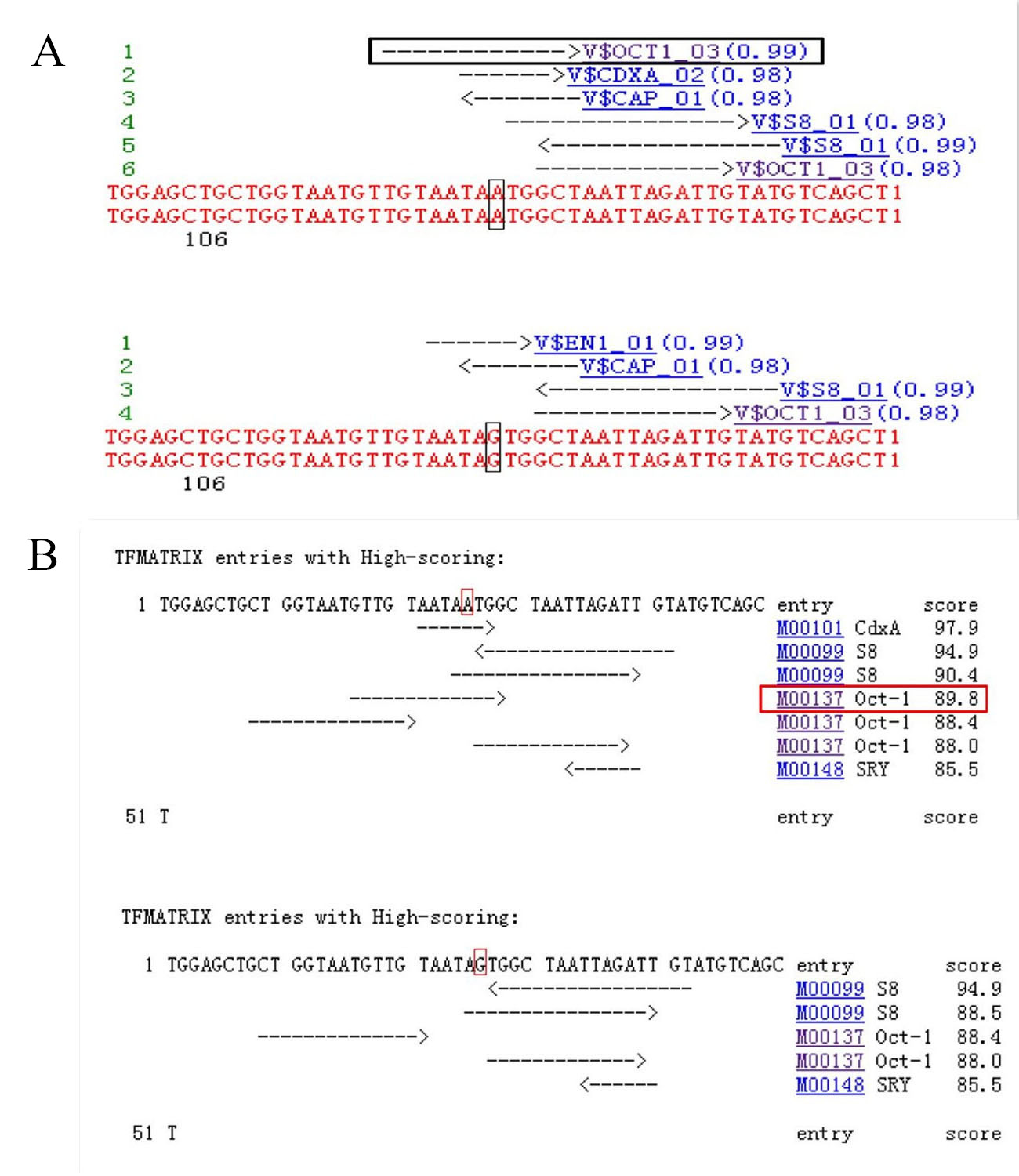

Supplement: Figure S1 — Quadrilateral frame indicated the substitutions and extra binding site of OCT-1. (A) Predicted by BIOBASE online software. (B) Predicted by TFserach online software. [file peerj-04-2186-s001.png]

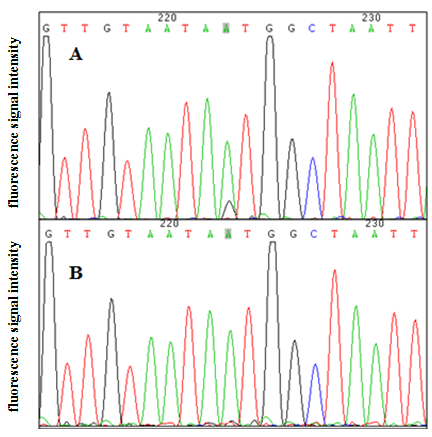

Supplement: Figure S2 — (A) PK cells. (B) ST cells. MARC0022311 was marked in gray backgound. [file peerj-04-2186-s002.png]

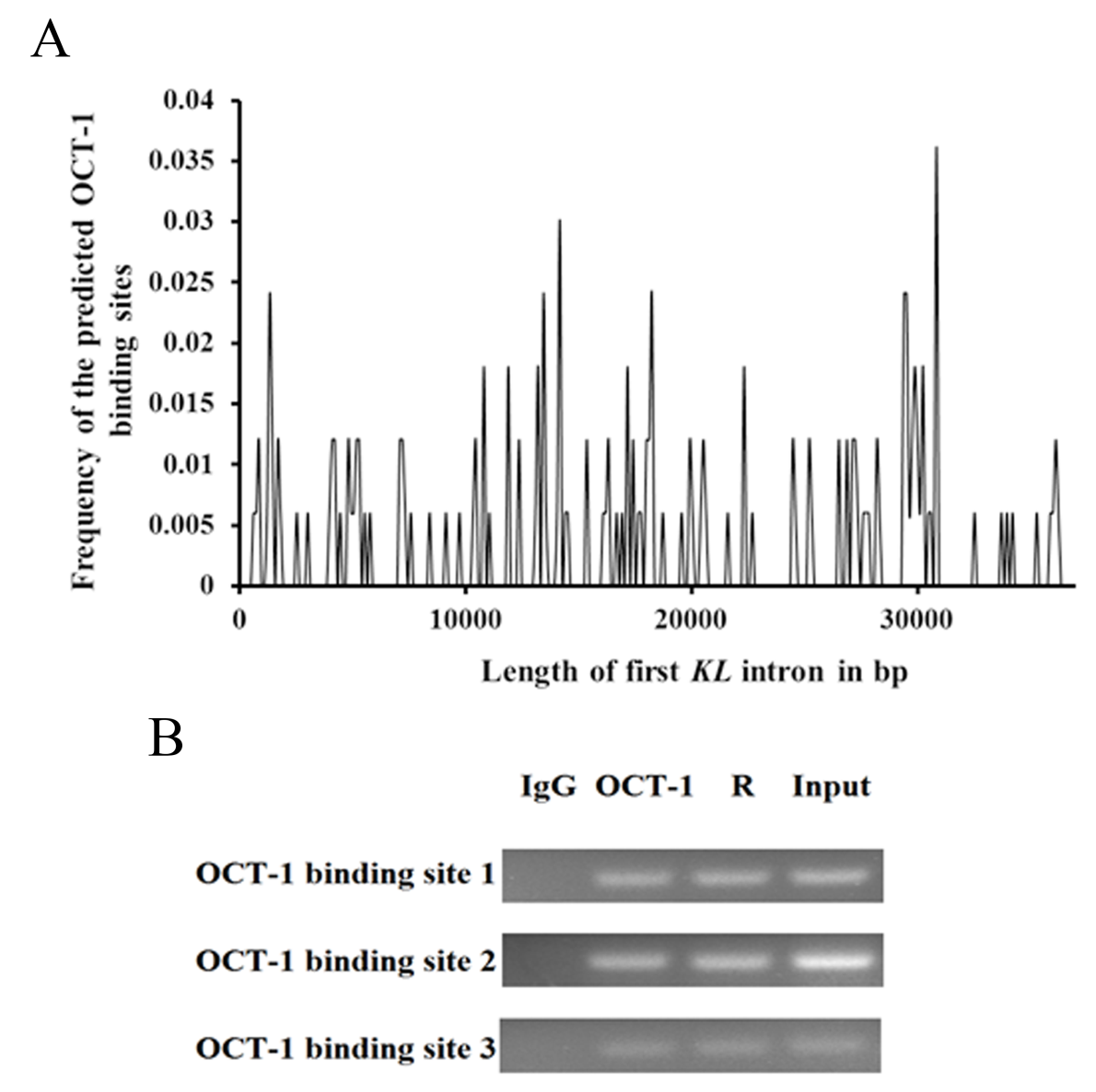

Supplement: Figure S3 — (A) Frequency distribution of the predicted OCT-1 binding sites. X-axis indicated the length of the porcine KL intron 1 in bp. Y-axis was the frequency of the predicted OCT-1 binding sites. (B) ChIP analysis of three candidate OCT-1 binding sites (1,395 bp to 1,525 bp, 14,322 bp to 14,436 bp, 30,970 bp to 31,141 bp) in KL intron 1 in PK cells. Primers used for ChIP-PCR was shown in Table 1. Input and R were positive control, while IgG was the negative control. [file peerj-04-2186-s003.png]
